# Karyotypic analysis and FISH mapping of microsatellite motifs reveal highly differentiated XX/XY sex chromosomes in the pink-tailed worm-lizard (*Aprasia parapulchella*, Pygopodidae, Squamata)

**DOI:** 10.1186/1755-8166-6-60

**Published:** 2013-12-18

**Authors:** Kazumi Matsubara, Theresa Knopp, Stephen D Sarre, Arthur Georges, Tariq Ezaz

**Affiliations:** 1 Institute for Applied Ecology, University of Canberra, Canberra ACT 2601, Australia

**Keywords:** Reptile, Sex chromosome differentiation, Y chromosome degeneration

## Abstract

**Background:**

The infraorder Gekkota is intriguing because it contains multiple chromosomal and environmental sex determination systems that vary even among closely related taxa. Here, we compare male and females karyotypes of the pink-tailed worm-lizard (*Aprasia parapulchella*), a small legless lizard belonging to the endemic Australian family Pygopodidae.

**Results:**

We applied comparative genomic hybridization to reveal an XX/XY sex chromosome system in which the Y chromosome is highly differentiated from the X in both gross morphology and DNA sequence. In addition, FISH mapping has revealed that two microsatellite repeat motifs, (AGAT)n and (AC)n, have been amplified multiple times on the Y chromosome.

**Conclusion:**

XY karyotypes are found in other pygopodids (*Delma inornata* and *Lialis burtonis*), suggesting that the common ancestor of Pygopodidae also had XY sex chromosomes. However, the morphology and size of the Y chromosomes are different among the three species, suggesting that the processes underlying the evolution of sex chromosomes in the Pygopodidae involved chromosome rearrangements and accumulation and amplification of repeats.

## Background

**Figure 1:**
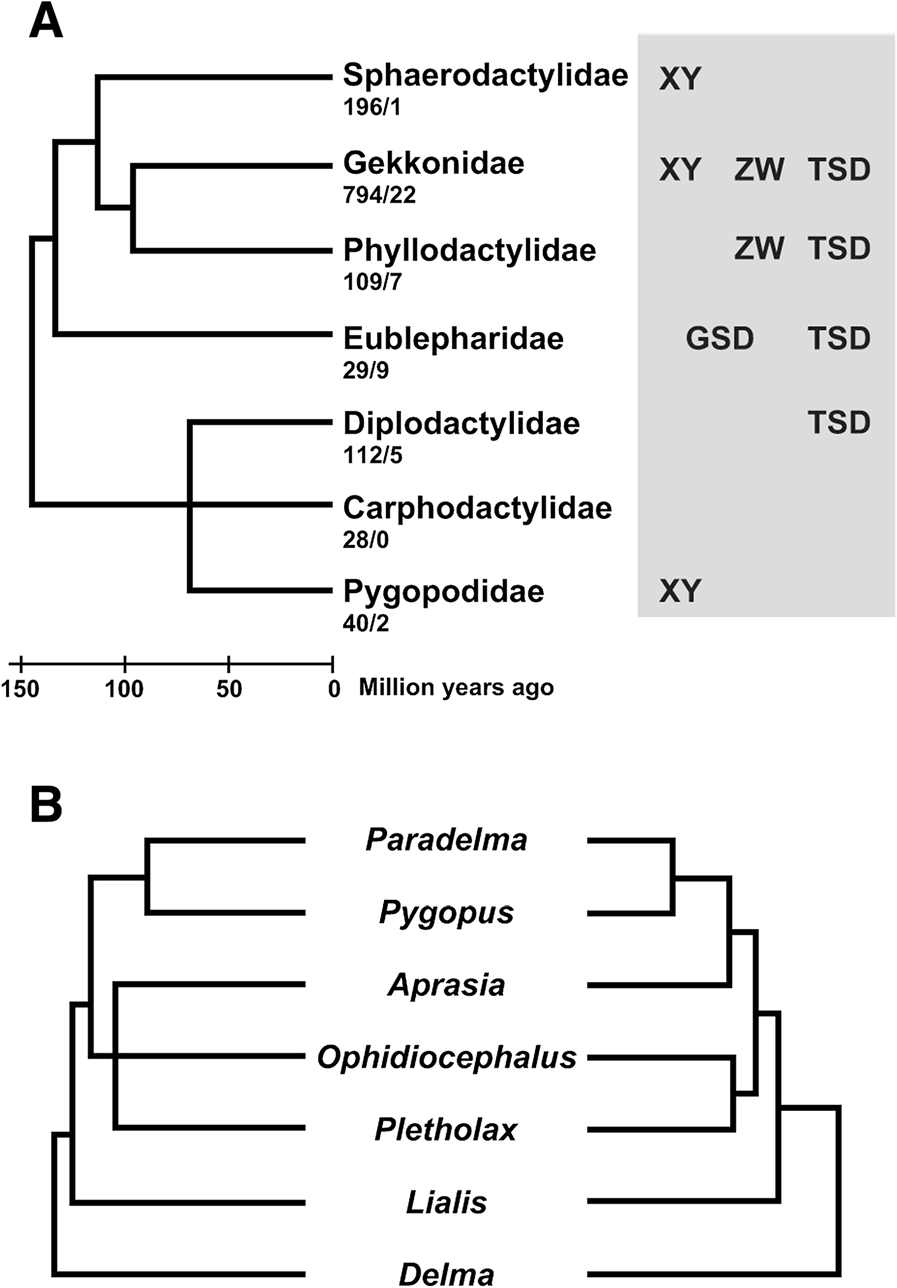
**Phylogenetic relationship and sex determination systems in Gekkota (A), and phylogenetic relationships in Pygopodidae (B). A**. Phylogenetic tree is referred to Vidal and Hedges [6]. The sex determination systems are adopted from Gamble [7]. The number of described species followed by the number of species with known sex determining mechanism is shown under each family. XY indicates clade contains species with male heterogamety, ZW indicates clade contains species with female heterogamety, TSD indicates clade contains species with temperature-dependent sex determination and GSD indicates clade contains species with genetic sex determination not associated with heteromorphic sex chromosomes. **B**. Phylogenetic trees of seven genera of Pygopodidae are adopted from Jennings et al. [8]: left and Oliver and Sanders [9]: right, respectively.

Sex determination in reptiles shows astonishing diversity in comparison with other amniotes. It includes temperature-dependent sex determination (TSD) in many turtles, all crocodiles, the tuatara and many lizards, a wide variety of female heterogamety and male heterogamety sex chromosomal forms including species with multiple sex chromosomes, and species in which temperature and sex chromosomes combine influence to determine sex [1-5]. Nowhere is this evolutionary lability more evident than that among the Gekkota, the oldest radiation of squamates. This infraorder includes species with XX/XY and ZZ/ZW modes of sex determination and TSD, with different sex determination forms even within the same family (Figure 1).

The Pygopodidae, endemic to Australia and New Guinea [10], is Gondwanan in origin and sits together with Australian geckos from the families Carphodactylidae and Diplodactylidae, within the infraorder Gekkota [6,9]. The three Australian Gekkota families diverged around 70 million years ago (Figure 1A). Species within Pygopodidae are known as flap-footed lizards or legless lizard because they have no forelimbs but retain vestigial hind limbs in the form of small scaly flaps [11]. The Family comprises seven genera – *Aprasia*, *Delma*, *Lialis*, *Ophidiocephalus*, *Paradelma, Pletholax* and *Pygopus*. Lineages represented today by *Delma* and then *Lialis* are the first genera to diverge from a common ancestor (Figure 1B) [8,9].

Pygopodids have chromosomes ranging in number from 2n = 34 in *Lialis burtonis* to 36 in *Delma inornata* and 38 in *D. fraseri, Ophidiocephalus spp.* and *Pygopus spp.*[12]. Only two species, *Delma inornata* and *Lialis burtonis*, have the direction of heterogamety determined, and both have male heterogamety [13,14]. However, there are some important differences between the two species. *D. inornata* has XX/XY sex chromosomes in which the submetacentric Y chromosome is much larger than acrocentric X chromosome [14], whereas *L. burtonis* has an X_1_X_1_X_2_X_2_/X_1_X_2_Y sex chromosome system with the Y chromosome being intermediate in size between the X_1_ and X_2_ chromosomes [13]. Based on the presence of male heterogamety in these two species, pygopodids are generally regarded to have male heterogamety [14], but this generalization is premature given that these two species represent only 2 out of seven pygopodid genera. Most chromosomal studies of this family have been equivocal on the sex chromosomes, so it is not yet clear that male heterogamety is the universal or even typical state.

The Pink-tailed worm-lizard, *Aprasia parapulchella*, is a small pygopodid [8,10,15] largely confined in distribution to the Australian Capital Territory (ACT) and a few remaining outlying populations in the New South Wales and Victoria [11]. At present, *A. parapulchella* is threatened with extinction through habitat loss caused by conversion to farmland or urban development. In a previous study [16] using 25 microsatellite loci, two loci, APP6 (Accession no. JQ713339 containing the microsatellite motifs CATT and GT) and APP40 (JQ713352 containing the microsatellite motif AGAT), showed sex specific polymorphisms. Each of the two loci was heterozygous in only 4 of 40 males (10%) but heterozygous in 33 of 69 females (50%) and 23 of 69 females (33.3.%), respectively. These two loci are likely to be linked to the sex chromosomes in this species.

In this study, we examined karyotype of *A. parapulchella* and identified the sex chromosome of this species using comparative genomic hybridization. In addition, we also mapped three microsatellite motifs – AGAT, AATG (reverse complement of CATT) and AC (reverse complement of GT) – to chromosomes using FISH and inferred the process of sex chromosome differentiation in this species.

## Results

### Karyotyping

**Figure 2:**
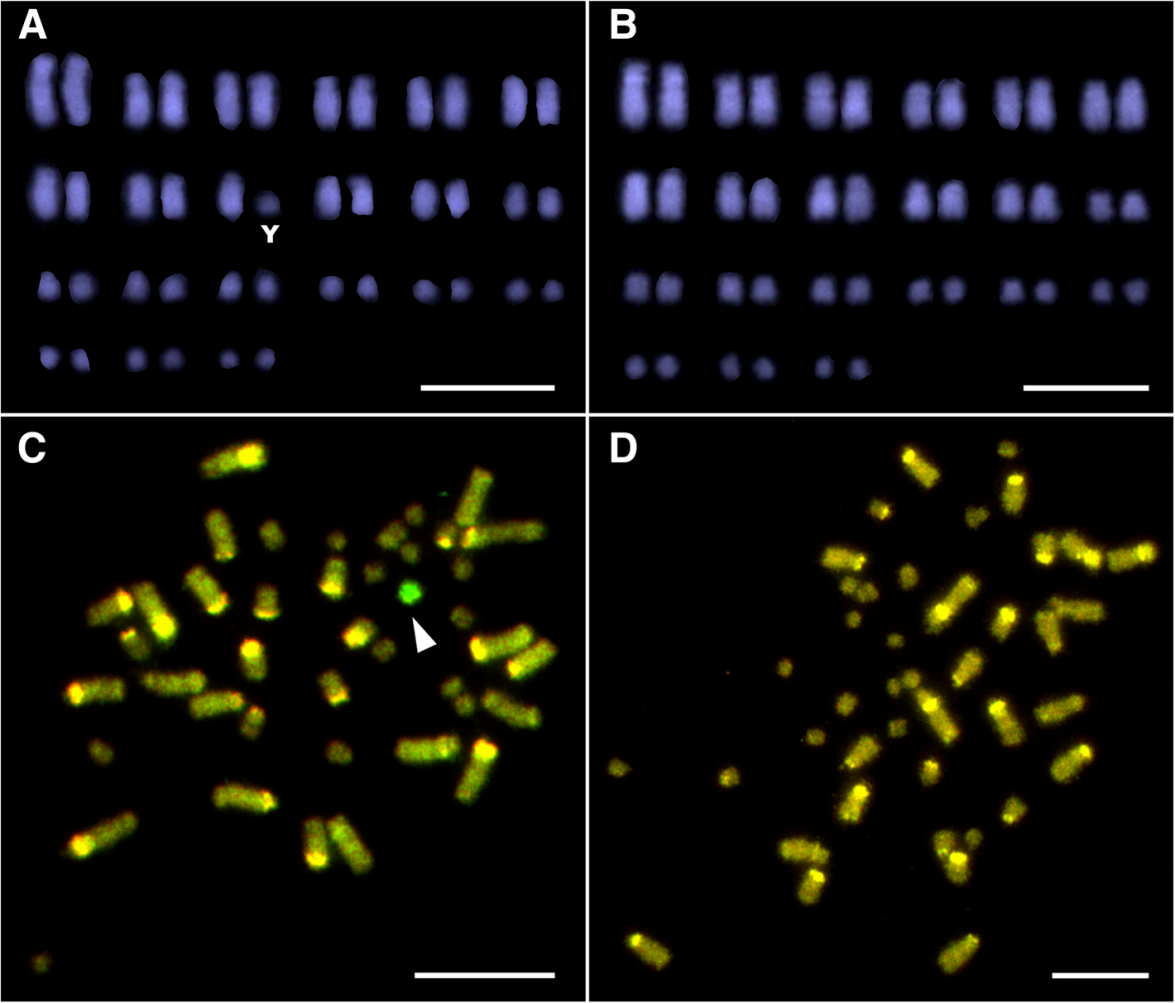
**DAPI-stained karyotypes and CGH images of male and female of *Aprasia parapulchella*.** DAPI-stained karyotypes of male **(A)** and female **(B)**, and CGH images in male **(C)** and female **(D)**. ‘Y’ and an arrowhead indicate Y chromosome **(A, C)**. Scale bars = 10 μm.

DAPI-staining of the karyotypes identified the diploid number of chromosomes for *A. parapulchella* to be 2n = 42 (Figure 2A, B). The chromosomes showed gradual variations in size so that there was no clear division into macro and microchromosomes, which is atypical for reptiles. Comparison of the karyotypes between males and females showed a heteromorphic pair in males (Figure 2A, B), indicating that this species has XY sex chromosomes. It was difficult to distinguish X and Y chromosomes from autosomes based on size and morphology alone, but they are mid-sized and small-sized chromosomes, respectively.

### Comparative genomic hybridization (CGH)

CGH images showed a bright hybridization signal on one of the small chromosomes in metaphase spreads in males but not in females (Figure 2C, D). This is further evidence that *A. parapulchella* has a XX/XY sex chromosomes, that the Y chromosome is smaller than the X. The X chromosome is not distinguishable from the autosomes by CGH.

### Fluorescence *in situ* hybridization (FISH) mapping of microsatellite motifs

**Figure 3:**
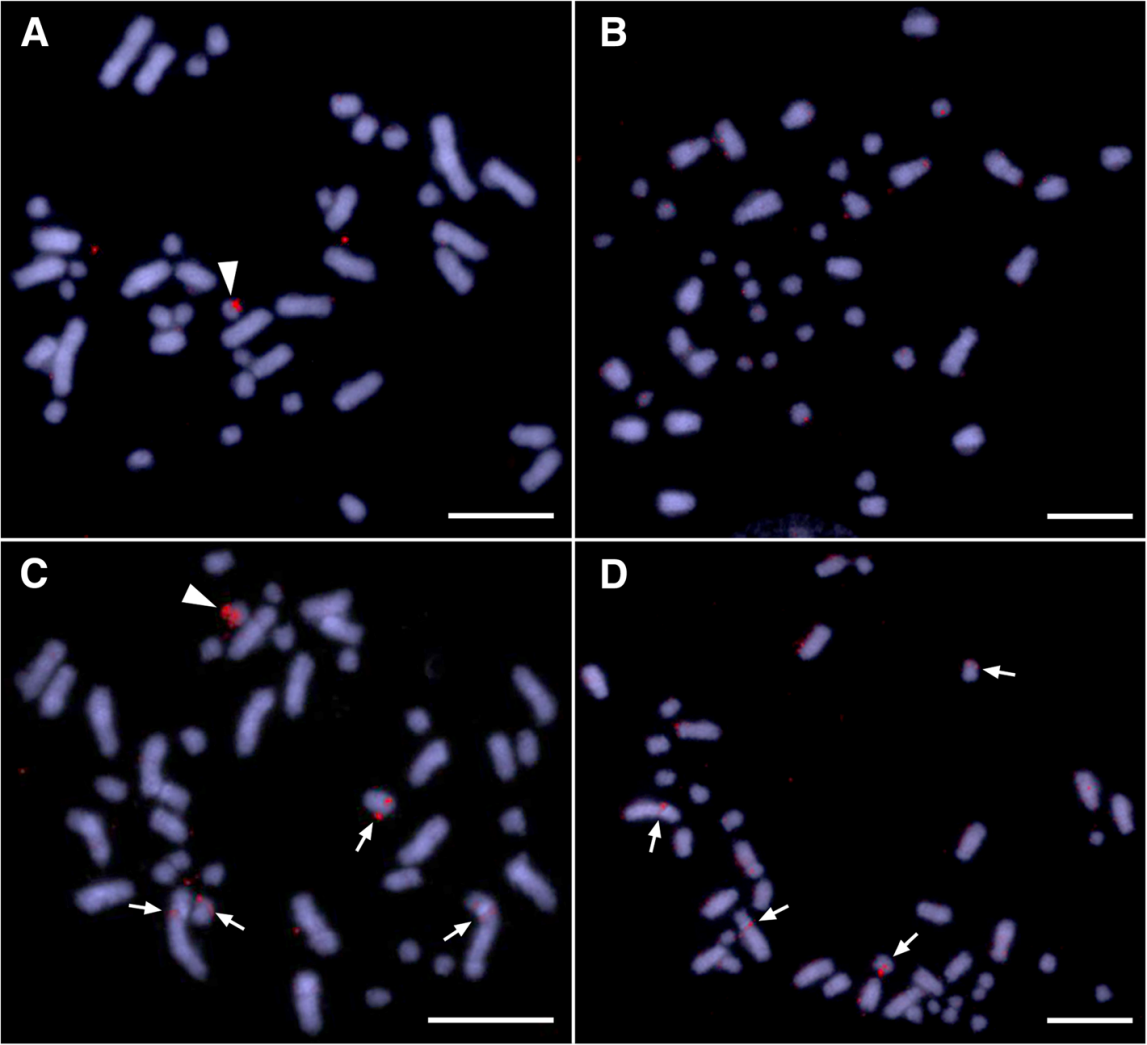
**FISH mapping of two microsatellite motifs in male and female of *A. parapulchella*.** FISH mapping of (AGAT)_8_ in male **(A)** and female **(B)**, and (AC)_15_ in male **(C)** and female **(D).** Arrowheads indicate hybridization signals on the Y chromosome **(A, C)**. Arrows indicate hybridization signals on two pair of autosomes **(C, D)**. Scale bars = 10 μm.

Three microsatellite motifs, (AATG)_8_, (AGAT)_8_, and (AC)_15_, were mapped by FISH on to the chromosomes of both sexes. The (AATG)_8_ probe showed no specific signal in either male or female metaphases (data not shown) whereas (AGAT)_8_ showed intense hybridization signal at the centromeric region of one small size chromosome in the male metaphase (Figure 3A), but not in the female metaphase (Figure 3B). This suggests that the AGAT microsatellite repeat has been amplified on the centromeric region of the Y chromosome in this species but not on the X. A bright and large hybridization signal from the (AC)_15_ motif was observed on a small chromosome in male metaphase but not in female metaphase (Figure 3C, D) whilst also being observed on one pair of small chromosomes and one pair of large chromosomes in both sexes (Figure 3C, D). We infer from this that the AC microsatellite repeat is also amplified on the Y chromosome and not on the X, with shared repeats in two other pairs of chromosomes.

## Discussion

Our analysis has demonstrated that the chromosome number of *A. parapulchella* is 2n = 42, the largest among all pygopodids thus far examined. The most common karyotype found among species of Diplodactylidae and Carphodactylidae is 38 acrocentric chromosomes [17], shared by pygopodid species of *Delma*, *Ophidiocephalus* and *Pygopus*, suggests that the chromosome number in the common ancestor of the Pygopodidae was also 38. It therefore appears likely that the chromosome number has increased in the lineage of *A. parapulchella* possibly through chromosome inversions and subsequent fissions.

The intense CGH signal on a single chromosome with the male genomic DNA reveals a Y chromosome that is highly differentiated from the X chromosome not only in morphology but also in DNA content. The Y chromosome of this species has therefore probably degenerated and accumulated large quantities of male specific DNA, much of which is likely to be repetitive [18,19]. Two microsatellite motifs, (AC)_15_ and (AGAT)_8_, were mapped with intense fluorescent signals on to the Y chromosome also suggest accumulation and amplification of repetitive sequences. The lengths of the microsatellite repeats in the two microsatellite loci, APP6 and APP40, are 16 bp and 40 bp (JQ713339, JQ713352) and FISH analysis can not localize accurately such short length of DNA fragments. Therefore, our FISH mapping did not identify exact location of the two loci. However, the results clearly indicate that the two microsatellite repeats are in high copy number on the Y chromosome, and that the two microsatellite repeats, (AGAT)n and (AC)n, form the main components of the centromeric region and the long arm of the Y chromosome, respectively. It is commonly thought that suppression of recombination between the sex chromosomes favors the accumulation of repetitive DNA sequences such as retrotransposons, ribosomal DNAs and microsatellite repeats on the non-recombining regions increasing the level of differentiation between sex chromosomes [20]. Such accumulation of repeats on sex chromosomes has been reported in many species of animals and plants (for example [21-30]), and, this process would also appear to have occurred in *A. parapulchella*.

**Figure 4:**
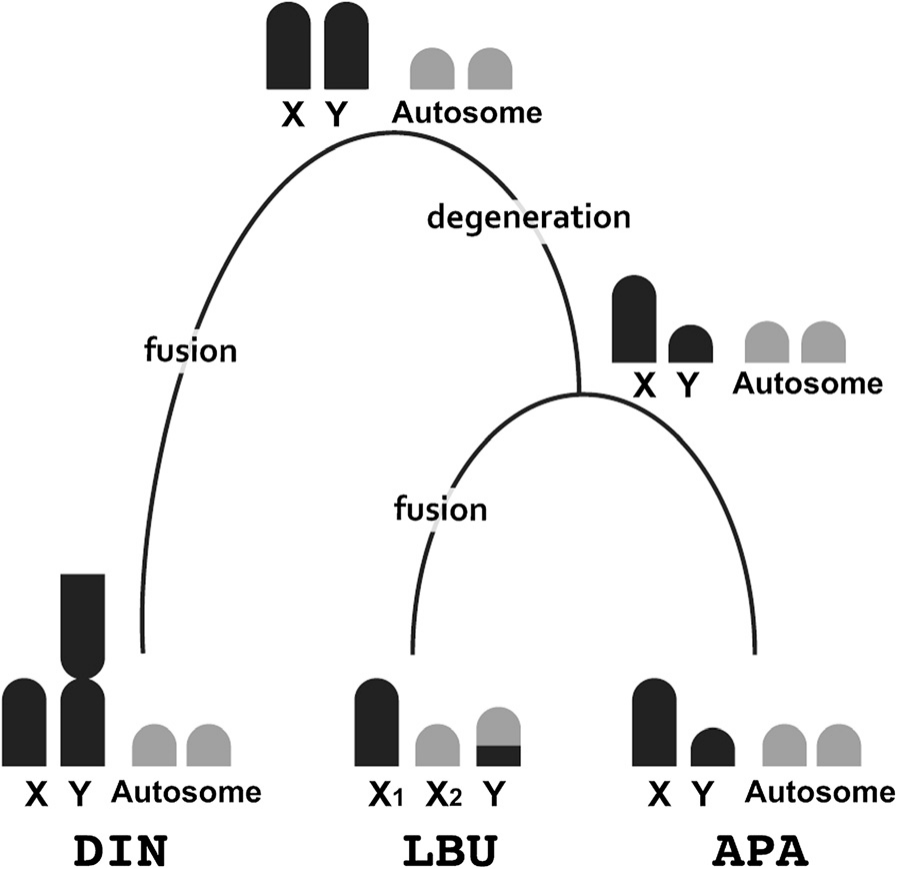
**Schematic model for sex chromosome evolution in the three pygopodids.** APA, LBU and DIN represent *Aprasia parapulchella*, *Lialis burtonis* and *Delma inornata*, respectively. Chromosome data of *L. burtonis* and *D. inornata* were obtained from Gorman and Gress [13] and King [14]. Phylogeny is modified after Jennings et al. [8] and Oliver and Sanders [9]. Possible chromosome rearrangements in sex chromosome evolution in the three pygopodids are shown on each of the branch.

The three pygopodids for which sex chromosomes have now been identified – *Aprasia parapulchella*, *Delma inornata*, and *Lialis burtonis* – exhibit male heterogamety. Given that these species come from three major clades within the Pygopodidae, including those representing basal lineages, it is likely that the common ancestor also had male heterogamety (Figure 4). The X chromosomes of *A. parapulchella* and *D. inornata* are morphologically similar, but their Y chromosomes differ in size and morphology. The Y chromosome in *A. parapulchella* is much smaller than the X chromosome, while the Y chromosome in *D. inornata* is submetacentric and much larger than the acrocentric X chromosome. Thus, differentiation of the Y chromosome has probably evolved independently in the *Aprasia* and *Delma* lineages. Moreover, the X_1_ of *L. burtonis* is larger than the X_2_ but similar in size and morphology to the X chromosomes of the other two species [13,14]. This suggests that the X_1_ of *L. burtonis* retains the original X chromosome inherited from the common ancestor of the Pygopodidae. If so, then the X_2_ of *L. burtonis* will have evolved from one of the smaller autosomes in the common ancestor, and a chromosome fusion is likely to have occurred between that autosome and the Y chromosome in the lineage of *L. burtonis* (Figure 4). Further study of the chromosome homology between the X_2_ and the part of Y chromosome will be required to confirm or refute this proposition that such fusion has occurred. It has been suggested that the large submetacentric Y chromosome of *D. inornata* formed by addition of a chromosome arm [14], but this too requires experimental verification.

## Conclusions

Taken together, our data build the case for an ancestral XY sex chromosome system in pygopodids. It is clear that like many other gekkonids, sex chromosomes in the pygopodids have a complex evolutionary history, with all three species examined so far differing from each other in key characteristics, such as sex chromosome sizes and morphologies. Although data are available from the limited number of species, it is likely that degeneration of Y chromosome have occurred in the common ancestor of *A. parapulchella* and *L. burtonis* and chromosome rearrangements such as fusion also played critical role in sex chromosome evolution in *D. inornata* and *L. burtonis*. Further experiments such as cross species chromosome painting using sex chromosomes, comparative mapping of sex chromosome-linked genes and repetitive sequences in several pygopodids and representative species from other gecko families are necessary to infer the sex chromosome evolution in Pygopodidae more precisely.

## Methods

### Animals

Tail tips cut from two male and two female Pink-tailed worm-lizards (*Aprasia parapulchella*) were collected from sites in the ACT for cell culture. Animal collection, handling, sampling and all other relevant procedures were performed following the guidelines of the Australian Capital Territory Animal Welfare Act 1992 (Section 40), and conducted under Permit LT2012587 (ACT Government) and CEAE 11/12 (the Committee for Ethics in Animal Experimentation at the University of Canberra). Two males and two females were used in the experiments.

### Chromosome preparation

Metaphase chromosome spreads were prepared from fibroblast cell lines of tail tissues following protocol described in Ezaz et al. [31]. Briefly, minced tail tissues were implanted in a T25 culture flask containing AmnioMax medium (Life Technologies, Carlsbad, California, USA) and were allowed to propagate under the condition of 28°C and 5% CO_2_. Once the fibroblast cells had grown to about 80% confluency, they were split into T75 flasks and subsequently split up to at least four passages before the chromosomes were harvested. Colcemid (Roche, Basel, Switzerland) was added to the culture flask at 75 ng/ml as the final concentration prior to harvesting. Following harvesting, cultured cells were fixed in 3:1 methanol:acetic acid and the cell suspension dropped onto glass slides, air-dried and stored at −80°C.

### DNA extraction and probes synthesis

Total genomic DNA was extracted from cultured fibroblasts using the DNeasy kit (Qiagen, Venlo, Netherlands) and following the manufacturer’s protocol. The oligonucleotides labeled with Cy3 of three microsatellite motifs, (AGAT)_8_, (AATG)_8_ and (AC)_15_, were purchased from GeneWorks (Hindmarsh, SA, Australia).

### Fluorescence *in situ* hybridization (FISH) with microsatellite motifs

FISH and CGH were conducted according to our previous study [32] with slight modification as follows. 500 ng of oligonucleotides of microsatellite motifs were mixed with 15 μl hybridization buffer (50% formamide, 10% dextran sulfate, 2xSSC, 40 mmol/L sodium phosphate pH7.0 and 1x Denhardt’s solution). The hybridization mixture was placed on a chromosome slide and sealed with a coverslip and rubber cement. The probe DNA and chromosome DNA were denatured by heating the slide on a heat plate at 68.5°C for 5 min. The slides were hybridized overnight in a humid chamber at 37°C. The slides were then washed by the following series; 0.4xSSC, 0.3% IGEPAL (Sigma-Aldrich, St. Louis, Missouri, USA) at room temperature for 2 min followed by 2xSSC, 0.1% IGEPAL at room temperature for 1 min. The slides were dehydrated by ethanol series and air-dried and then counterstained using 20 μg/ml DAPI, 2xSSC and mounted with anti-fade medium, Vectashield (Vector Laboratories, Burlingame, California, USA). FISH images were captured using a Zeiss Axioplan epifluorescence microscope equipped with a CCD (charge-coupled device) camera (Zeiss, Oberkochen, Germany). ISIS software or AxioVision (Zeiss) was used for microphotography and analyzing images.

### Comparative genomic hybridization (CGH)

Genomic DNA was labelled by nick translation incorporating SpectrumGreen-dUTP (Abbott, North Chicago, Illinois, USA) for males and SpectrumOrange-dUTP (Abbott) for females. The labelled male and female DNA was coprecipitated with 20 μg glycogen as carrier, and dissolved in 15 μl hybridization buffer. The hybridization and washes were carried out as above with slight modification. Specifically, hybridization was carried out for 3 days and the slides were washed by 0.4xSSC, 0.3% IGEPAL at 55°C for 2 min and then 2xSSC, 0.1% IGEPAL at room temperature for 1 min.

## Abbreviations

TSD: Temperature-dependent sex determination; CGH: Comparative genomic hybridization; FISH: Fluorescence *in situ* hybridization; SSC: Saline sodium citrate; DAPI: 4′,6-diamidino-2-phenylindole; CCD: Charge-coupled device; dUTP: Deoxyuridine triphosphate; GSD: Genotypic sex determination.

## Competing interests

The authors declare that they have no competing interests.

## Authors’ contributions

The authors have made the following declarations about their contributions. TE and KM conceived, designed and directed the experiments. KM performed the experiments. KM wrote the first draft and all coauthors contributed in reviewing the paper. All authors read and approved the final manuscript.
